# Hydrous oceanic crust hosts megathrust creep at low shear stresses

**DOI:** 10.1126/sciadv.aba1529

**Published:** 2020-05-27

**Authors:** Christopher J. Tulley, Åke Fagereng, Kohtaro Ujiie

**Affiliations:** 1 School of Earth and Ocean Sciences, Cardiff University, Cardiff, UK.; 2 Graduate School of Life and Environmental Sciences, University of Tsukuba, Tsukuba, Japan.

## Abstract

The rheology of the metamorphosed oceanic crust may be a critical control on megathrust strength and deformation style. However, little is known about the strength and deformation style of metamorphosed basalt. Exhumed megathrust shear zones exposed on Kyushu, SW Japan, contain hydrous metabasalts deformed at temperatures between ~300° and ~500°C, spanning the inferred temperature-controlled seismic-aseismic transition. Field and microstructural observations of these shear zones, combined with quartz grain-size piezometry, indicate that metabasalts creep at shear stresses <100 MPa at ~370°C and at shear stresses <30 MPa at ~500°C. These values are much lower than those suggested by viscous flow laws for basalt. The implication is that relatively weak, hydrous, metamorphosed oceanic crust can creep at low viscosities over a wide shear zone and have a critical influence on plate interface strength and deformation style around the seismic-aseismic transition.

## Teaser

Hydration allows subducting oceanic crust to be far weaker than previously recognized.

## INTRODUCTION

Along convergent margins, a kilometers-thick shear zone typically separates the relatively rigid subducting and overriding plates (*1*). A much-used conceptual model is that the top of the mechanically strong basaltic oceanic crust is lubricated by weak subducting sediments (*2*–*4*), allowing the plate interface to creep at low shear stresses. However, the thickness of subducting sediment is variable within and between margins, and during subduction, mineral dehydration and compaction may contribute to volume reduction >50% by the time sediments have been buried to >10 km (*5*, *6*). Subducting oceanic crust is also topographically rough in many creeping margins, meaning that creep occurs despite the presence of seamounts and horsts with thin sediment cover (*7*). Therefore, although seismic observations commonly suggest the presence of a kilometers-thick creeping layer at the top of deeply subducted oceanic lithosphere (*1*, *8*), the oceanic crust may be exposed to the overriding plate along sediment-starved margins and in areas with thin to no sediment coverage. Creep of the oceanic crust would negate the need for a continuous layer of deeply subducted lubricating sediments, but it is unclear whether oceanic crust can be sufficiently weak to accommodate plate-rate creep at low inferred shear stresses.

Fresh basaltic rocks have high frictional strengths (0.5 ≤ μ_f_ ≤ 0.75) and display velocity-weakening behavior (*9*), a requirement for earthquake nucleation. Viscous creep of dry basalt at shear stresses <200 MPa requires temperatures >650°C, assuming a moderate strain rate (10^−12^ s^−1^) (*10*). Such high strengths are incompatible with the inferred low shear strengths of creeping plate interfaces at the base of the seismogenic zone (*11*). However, phyllosilicate minerals that form during metamorphism of hydrated oceanic crust at greenschist, blueschist, and amphibolite facies conditions (*12*) deform at low shear stresses and are velocity strengthening over a wide pressure-temperature (P-T) range (*13*, *14*). The transition from blueschist or amphibolite to eclogite is expected to notably increase the viscosity of oceanic crust (*3*); however, here, we are considering sub-eclogite facies conditions.

We investigate the hypothesis that hydrous, metamorphosed oceanic crust with a weak phyllosilicate-bearing mineralogy may creep at shear stresses low enough to accommodate significant plate interface displacement at geological strain rates. Field and microstructural observations from exhumed megathrust shear zones exposed at three localities on Kyushu, southwest (SW) Japan, indicate that, during warm subduction, hydrated oceanic crust is far weaker than dry basalt and comparable in strength to metasediment. We conclude that hydrous oceanic crust may accommodate significant megathrust shear strain and therefore may have a strong influence on the strength and deformation style of the plate interface.

### Geological background

**Fig. 1:**
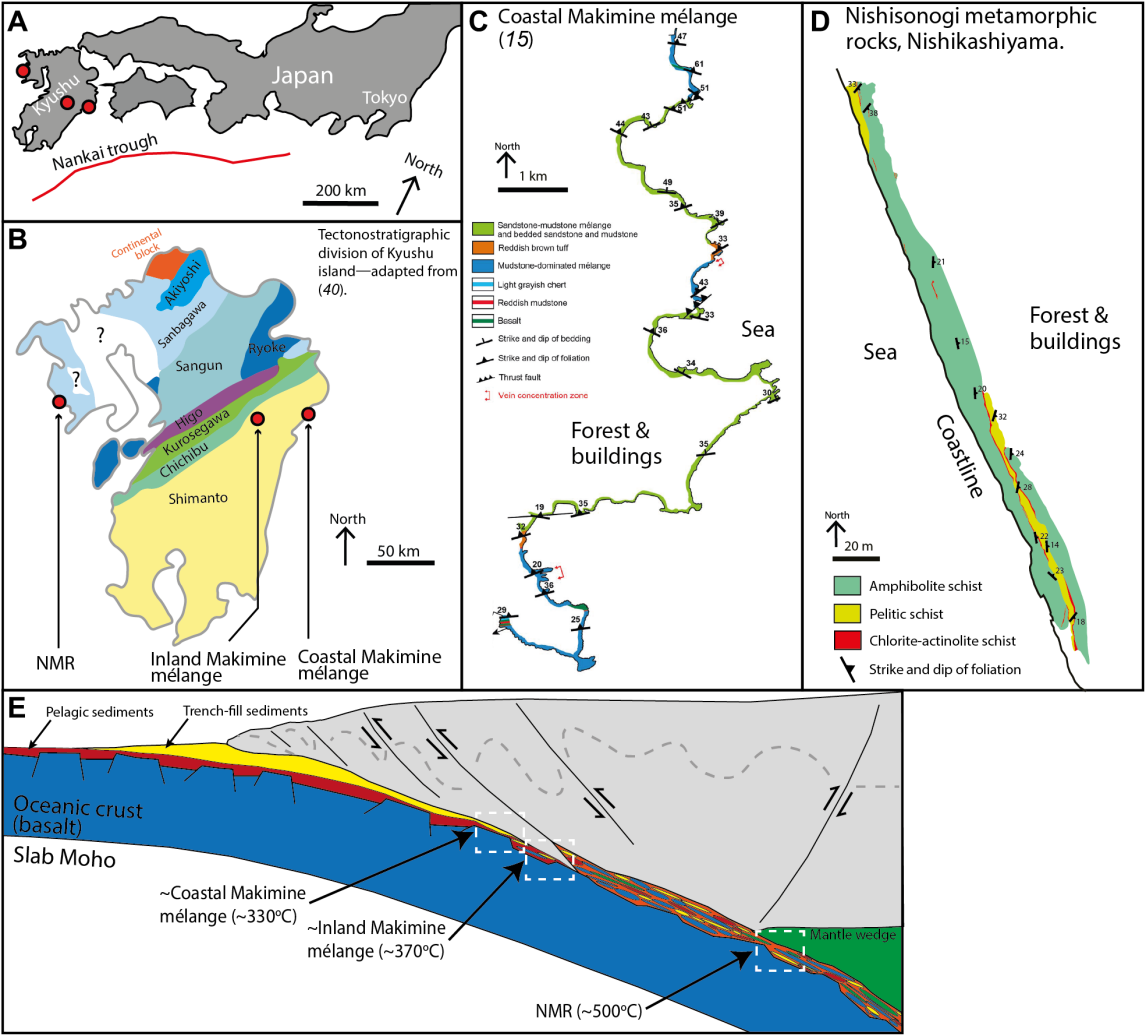
Geological background and geotectonic setting. (**A**) Current tectonic setting and location of Kyushu island. (**B**) Simplified map of accretionary terranes and high P-T metamorphic belts on Kyushu island after (*43*). (**C**) Geological map of Coastal Makimine mélange (*15*). (**D**) Geological map of a coastal exposure of the NMR. (**E**) Cartoon cross section of a generic convergent margin showing approximate settings of exhumed shear zones.

The Shimanto accretionary complex and Nishisonogi metamorphic rocks (NMR) represent oceanic crust and metasediments emplaced along the eastern margin of the Eurasian continent in the Late Cretaceous (Fig. 1). The Makimine mélange occurs within the Shimanto accretionary complex and preserves megathrust deformation structures in metabasalt, mudstone, sandstone, and tuff deposits (Fig. 1C) (*15*). The NMR preserves deformation in amphibolite schist (metabasalt), pelitic schist, and rare serpentinite bodies (Fig. 1D) (*16*).

Cooling ages for the Shimanto accretionary complex suggest that exhumation of the Makimine mélange occurred in the Late Cretaceous (*17*). The NMR are thought to have experienced Late Cretaceous metamorphism (*18*). Tectonic reconstructions suggest that, during the Late Cretaceous, young to moderately aged oceanic lithosphere subducted beneath northeast (NE) Asia (*19*). On this basis, we interpret that our observations from the Makimine mélange and the NMR represent plate interface deformation during the subduction of young to moderately aged oceanic lithosphere (which is therefore warm and buoyant) toward the north-northwest (NNW) along a NE-SW striking paleo-Pacific margin.

In the coastal Makimine mélange (exposed near 32.720806°N, 131.855900°E), oceanic plate stratigraphy is generally preserved. Lenses of greenish metabasalt and hemipelagic red mudstone are overlain by reddish-brown tuff, in turn overlain by sandstone-mudstone mélange and coherent turbidites (Fig. 1C) (*15*). This stratigraphic sequence is repeated at least twice perhaps because of underplating (*15*). The mélange accommodated thrust-sense deformation by viscous solution-precipitation creep and brittle displacements reflected by a quartz-filled fault-fracture mesh (*15*). Peak metamorphic temperature is estimated to be 328 ± 30°C based on Raman spectra of carbonaceous material (RSCM) (*15*); furthermore, the vein quartz is not dynamically recrystallized, consistent with deformation at temperatures <350°C (*20*).

Inland Makimine mélange exposures (near 32.720806°N, 131.855900°E) show meter to centimeter thick, mostly continuous layers of metabasalt and metasediment. RSCM suggests peak metamorphic temperatures of 371 ± 19°C (*21*). RSCM temperature estimates from coastal and inland mélange exposures are within the range suggested for the Shimanto accretionary complex, on the basis of prehnite-actinolite to greenschist facies mineral assemblages and earlier studies using vitrinite reflectance, illite crystallinity, and RSCM thermometers (*17*, *22*).

The NMR are interpreted to represent a plate interface shear zone exhumed from epidote-blueschist facies conditions (*16*). There is no indication of preserved ocean plate stratigraphy in the NMR; rather, amphibolite schist and pelitic schist are interlayered in exposures of a broad kilometer-scale shear zone (Fig. 1D). RSCM temperature estimates suggest peak metamorphic temperatures of 500 ± 37°C (*21*), consistent with epidote-blueschist facies metamorphism and previous temperature estimates (*16*).

## RESULTS

### Coastal Makimine mélange

**Fig. 2:**
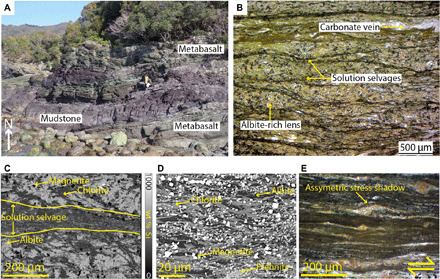
Outcrop and microscale appearance of metabasalts in coastal Makimine mélange. (**A**) Metabasalt exposed as foliated layers intercalated with red mudstone. (**B**) Photomicrograph of anastomosing solution selvages, which define a poorly developed foliation. (**C**) EDS element map for Si highlights reduction in Si concentration within solution seams, caused by dissolution of albite. (**D**) Backscattered electron image of solution selvages, which consist of very fine (mostly <10 μm) grains of chlorite, prehnite, and magnetite. (**E**) Photomicrograph of asymmetric stress shadows around titanite, indicating noncoaxial shear within solution selvages. Photo credit (A): Å. Fagereng, Cardiff University.

Foliation in the mélange dips shallowly to the NNW, and long axes of sandstone clasts in the mélange and stretching lineations (defined by elongate albite) measured on foliation planes plunge shallowly to the NNW (fig. S1), consistent with the inferred top-to-the south shear along the Cretaceous NE-SW striking margin. At the thin-section scale, metabasalt lenses contain albite-poor solution selvages, which define a poorly developed anastomosing foliation separating relatively albite-rich lenses (Fig. 2B). The typical spacing between solution selvages is 250 μm, and the thickness of each selvage ranges from 10 to 100 μm. Si concentration maps derived by energy-dispersive spectroscopy (EDS) highlight reduced albite content within solution selvages, relative to the albite-rich lenses (Fig. 2C). Backscattered electron images of solution selvages (Fig. 2D) show a well-developed microscale foliation defined by aligned chlorite, with interstitial prehnite and magnetite. Albite occurs within the selvages as grains that are elongate parallel to foliation.

### Inland Makimine mélange

**Fig. 3:**
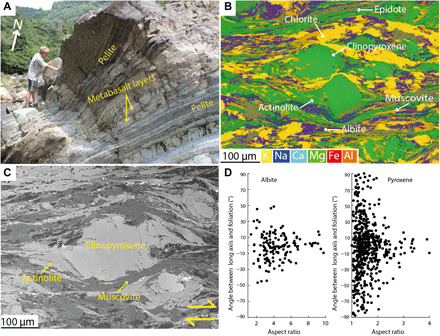
Outcrop and microscale appearance of metabasalts in inland Makimine mélange. (**A**) Metabasalt layers with low amplitude, long wavelength pinch, and swell geometry interlayered with metasediment. (**B**) EDS element map of inland Makimine metabasalt, where actinolite and chlorite form in stress shadows adjacent to clinopyroxene grains. (**C**) Backscattered electron image of actinolite in an asymmetric stress shadow about a clinopyroxene grain. (**D**) Albite and clinopyroxene grains have an SPO with long axes parallel to foliation, more pronounced in larger aspect ratio grains. Photo credit (A): C. Tulley, Cardiff University.

Foliation and lineation orientations within both metasediment and metabasalt are subparallel to those in the coastal Makimine mélange (fig. S1). Metabasalt layers have structural thicknesses of a few centimeters to a meter and display a low-amplitude, long-wavelength pinch and swell geometry (Fig. 3A). Quartz-filled foliation-parallel to oblique veins are ductilely deformed, forming boudins and layers with pinch-and-swell geometry along foliation planes (fig. S2). Microstructures in the quartz veins include subgrains and bulging grain boundaries, which indicate dynamic recrystallization of quartz, consistent with RSCM temperature >350°C (*20*), warmer than estimates for coastal Makimine mélange. Electron backscatter diffraction (EBSD)–derived pole figures for quartz show *c* axes distributed in a cross-girdle structure, indicative of a combination of basal <a> and prism <a> slip (fig. S2) (*23*). The foliation in metabasalt is defined by aligned actinolite, chlorite, and muscovite. Clinopyroxene grains have a weak shape preferred orientation (SPO) with grain long axes parallel to foliation (Fig. 3D). EBSD analysis of clinopyroxene indicates a weak crystallographic preferred orientation (CPO) with [001] aligned to the foliation, lattice distortions across clinopyroxene grains are generally <2^o^ (fig. S2), and there is no indication of dynamic recrystallization. Actinolite and minor chlorite form asymmetric stress shadows adjacent to clinopyroxene grains (Fig. 3, B and C). Small (<50 μm) albite grains are dispersed throughout the metabasalt and also have a strong SPO with long axes parallel to foliation (Fig. 3D).

### Nishisonogi metamorphic rocks

**Fig. 4:**
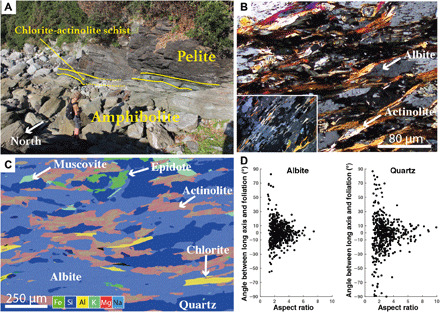
Outcrop and microscale appearance of amphibolite schist (metabasalt) in the NMR. (**A**) Amphibolite schist and pelitic schist separated by a thin (<1 m) layer of chlorite-actinolite schist. (**B**) In thin section, prolate grains of albite are enveloped by chlorite, muscovite, and actinolite, with an asymmetry showing a dextral sense of shear in the provided photomicrograph. Inset shows oriented inclusions of actinolite within albite and quartz. (**C**) EDS element map of amphibolite. Albite shows no chemical zonation, but actinolite has pronounced zoning with less aluminous composition in the rims. (**D**) Albite and dispersed quartz grains have a strong SPO with long axes parallel to lineation. Grains with larger aspect ratios are generally more closely aligned to the foliation. Photo credit (A): C. Tulley, Cardiff University.

Foliations in amphibolite schist and pelitic schist dip gently to the east, and stretching lineation plunges gently to the southeast (fig. S1). Thin (<0.5 m) layers of chlorite-actinolite schist cut the amphibolite and metapelite (Figs. 1D and 4A) and contain S-C structures and C plane lineations, which suggest top to the south noncoaxial shear (fig. S1). Quartz-filled foliation-parallel to oblique veins cut both the amphibolite and the pelite and are commonly boudinaged along the foliation (fig. S3). The eastward dip of the foliation in the NMR is interpreted to arise from post-subduction regional-scale folding with a north-south trending fold hinge (*16*). Removing the effects of this folding by restoring foliation to horizontal results in an approximately top-south shear sense, consistent with the inferred megathrust shear along the Cretaceous margin (*19*).

**Fig. 5:**
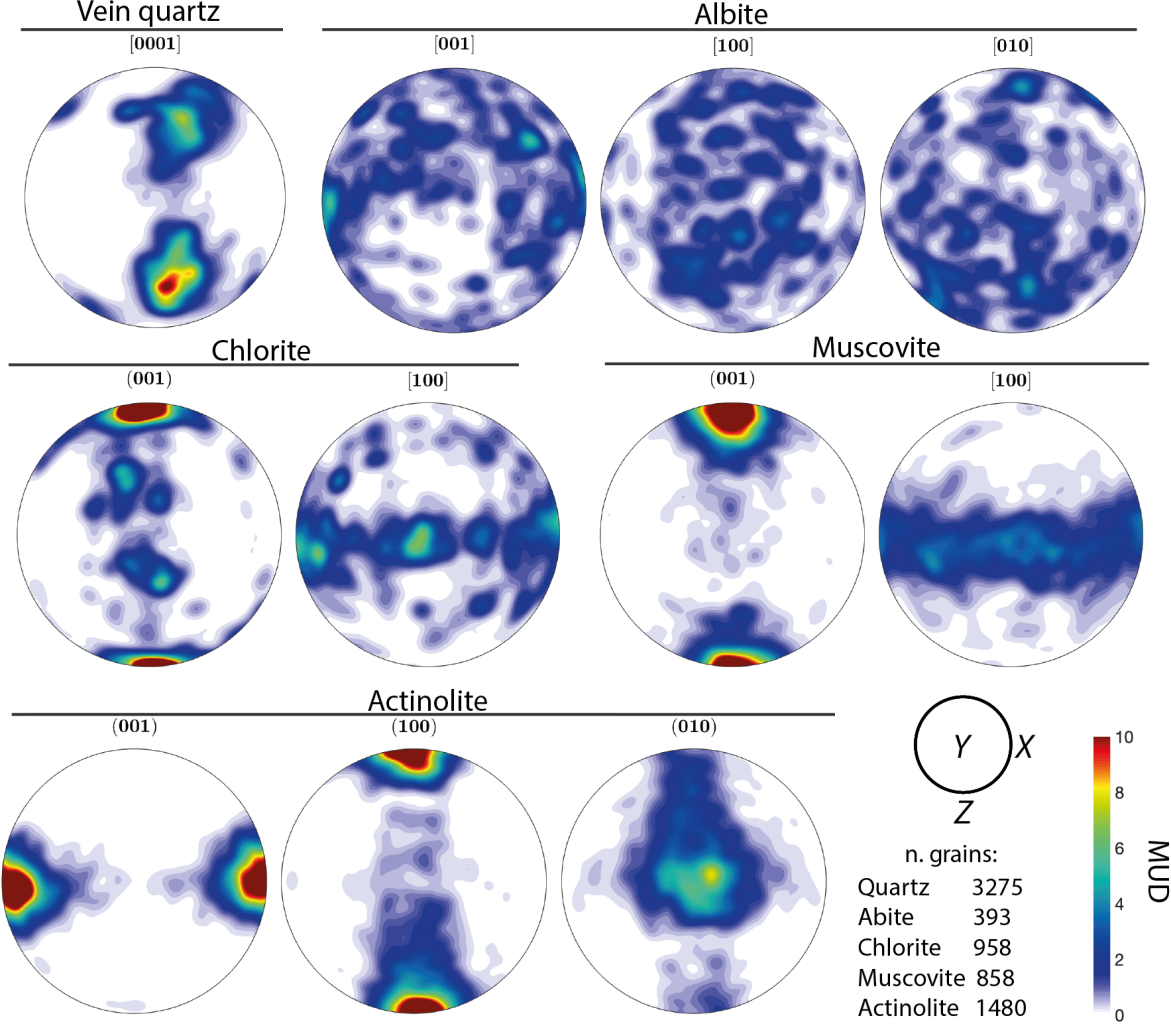
Pole figures (equal area, lower hemisphere) for grain orientations in metabasalt from the NMR. Contours in units of multiples of a uniform distribution (MUD); regions with MUD > 10 are shown in dark red. Quartz (boudinaged veins) shows two clusters each inclined between the *Y* and *Z* kinematic directions. Albite shows no clear preferred orientation. Muscovite and chlorite have strong preferred orientations with (001) planes parallel to foliation. Muscovite [100] forms a continuous girdle parallel to foliation, whereas in chlorite [100] is weakly clustered parallel and perpendicular to the shear direction. Actinolite [001] axes are aligned with the shear direction, and (100) and (101) planes lie parallel and orthogonal to foliation, respectively.

The long axes of albite and actinolite grains define the stretching lineation in amphibolite schist, whereas aligned muscovite and chlorite platelets define the foliation. Minor epidote, titanite, and apatite also occur in the metabasalt. Quartz is present in boudinaged veins and as dispersed single grains (Fig. 4C). Pole figures for muscovite and chlorite show a strong CPO consistent with the alignment observed in thin section. Both muscovite and chlorite have the common phyllosilicate preferred orientation (*24*) with (001) parallel to foliation and [100] distributed in a girdle along the foliation plane (Fig. 5). Pole figures indicate that some chlorite grains have (001) oriented nearly orthogonal to the foliation; these grains occur in stress shadows adjacent to albite grains.

Actinolite has a strong CPO with [001] parallel to lineation and (100) parallel to foliation (Fig. 5). There are, however, no microstructural indicators (such as subgrains or bulging grain boundaries) of dislocation creep in actinolite. EDS maps of actinolite show chemical zoning (Fig. 4C), where the cores of actinolite grains typically have a more aluminous, hornblende-like composition, which changes to a less aluminous, more actinolite-like composition toward the rims. In contrast to the hydrous minerals in the metabasalt, albite has no clear CPO (Fig. 5) but does have a strong SPO with long axes parallel to lineation (Fig. 4D). Boudinaged quartz veins (fig. S3) have a strong CPO, with two *c*-axis clusters inclined between the *Y* and *Z* kinematic directions (Fig. 5), indicative of rhomb <a> slip (*23*).

## DISCUSSION

### Fabric-forming mechanisms

The foliation observed in coastal Makimine metabasalt corresponds to alternating albite-poor and albite-rich layering (Fig. 2C), interpreted to represent local dissolution of albite along planes orthogonal to the greatest principal compressive stress (σ_1_). Localized dissolution of albite produced relative increases in chlorite along dissolution seams. In inland Makimine mélange, the SPO of clinopyroxene and albite also indicates pressure solution, again accommodating shortening orthogonal to σ_1_ (Fig. 3D). Aligned actinolite and chlorite form asymmetric stress shadows adjacent to clinopyroxene porphyroclasts and are interpreted as replacement textures that grew during noncoaxial shear (Fig. 3B). Overall, the fabric in both coastal and inland Makimine metabasalts is interpreted to be the product of shortening by dissolution-precipitation creep, coupled to noncoaxial shear by frictional sliding along foliae. The SPO of albite, actinolite, and dispersed quartz grains in NMR amphibolite indicates foliation-normal shortening associated with grain growth in preferred orientations (Fig. 4, C and D). The CPO and SPO of chlorite and muscovite (Figs. 4C and 5) are interpreted to have formed by rotation and growth of the phyllosilicates into mechanically stable orientations during shortening and noncoaxial shear at water-saturated metamorphic conditions favoring the stability of these phyllosilicates. The zoned actinolite (Fig. 4C) is interpreted to represent exhumation through the hornblende to actinolite reaction, expected between ~460°C and 500°C in mafic rocks (*12*), near the peak temperature conditions of this shear zone.

### Frictional-viscous creep

In the coastal Makimine mélange metabasalt, the formation of a solution cleavage gives rise to a mechanical anisotropy, given the contrasting frictional strengths of albite and foliated chlorite (μ_f_ = 0.6 and μ_f_ = 0.2, respectively) (*9*, *13*). In solution selvages, asymmetric stress shadows form adjacent to titanite grains (Fig. 2E), indicating that noncoaxial shear occurred in these layers.

In the inland Makimine metabasalt, the chlorite, actinolite, and epidote foliation is locally disrupted by rigid clinopyroxene grains, giving rise to a curvi-planar foliation at the microscale (Fig. 3B). SPO in albite and clinopyroxene, and the formation of actinolite and chlorite in stress shadows adjacent to clinopyroxene, suggests that deformation of the metabasalt occurred at least partially by dissolution-precipitation creep. Overall, deformation is interpreted to have occurred by frictional sliding along the actinolite, chlorite, and muscovite foliation, coupled to dissolution-precipitation creep of albite and clinopyroxene. The importance of dissolution-precipitation creep is consistent with previous observations of high-pressure, low-temperature metamorphic rocks (*25*).

In NMR amphibolite, slip along the phyllosilicate foliae is likely to be an important deformation mechanism, given the contrast between strong albite and the weak phyllosilicate minerals (*9*, *13*). The CPO of muscovite and chlorite (Fig. 5) reflects easy slip along (001) in phyllosilicates compared to slip along other crystallographic planes (*14*). Mechanically strong albite grains are commonly surrounded by the phyllosilicates and amphibole (Fig. 4B), suggesting that albite is a strong phase in a relatively weak phyllosilicate-dominated matrix. Albite grains have a strong SPO indicative of foliation-normal shortening (Fig. 4D). Crystallographic preferred orientations in albite do not correspond to recognized slip systems, suggesting deformation by a mechanism other than dislocation creep. Grains of actinolite, muscovite, and chlorite commonly occur as inclusions within albite and almost always have the same orientation as isolated grains (Fig. 4B), implying that albite grain boundaries moved diffusively around the hydrous minerals. These observations together strongly suggest that albite is deformed by diffusion, despite having a grain size much larger than is usually reported for diffusion creep (*25*, *26*). One possibility to explain the large grain sizes in albite is grain growth, stimulated by Na-rich fluids inferred to be present at peak metamorphic conditions in the NMR (*27*). Given the slow strain rates that can be accommodated by diffusion creep in albite at Nishisonogi-like conditions and grain sizes (*28*) and the relatively weak strength of (001) planes in phyllosilicates (*13*, *14*), we expect that albite accommodated minor amounts of strain relative to the phyllosilicates.

### Formation and deformation of quartz veins

Ductilely deformed quartz veins aligned with a foliation implies that the veins experienced deformation at the foliation-forming conditions, and their microstructure reflects these conditions. Assuming the veins formed during subduction, they also imply the presence of a silica-saturated fluid within subducting oceanic crust. Similarly, the presence of hydrous, fabric-forming minerals in all three shear zones also implies water-saturated conditions during deformation. Mineral dehydration reactions occurring at specific areas in P-T space are thought to supply fluid to the megathrust (*5*, *12*). The development of quartz-filled fractures in the creeping shear zones may be driven by a combination of local, transient increases in fluid pressure (*12*) and/or local stress amplifications during mélange deformation (*29*).

**Fig. 6:**
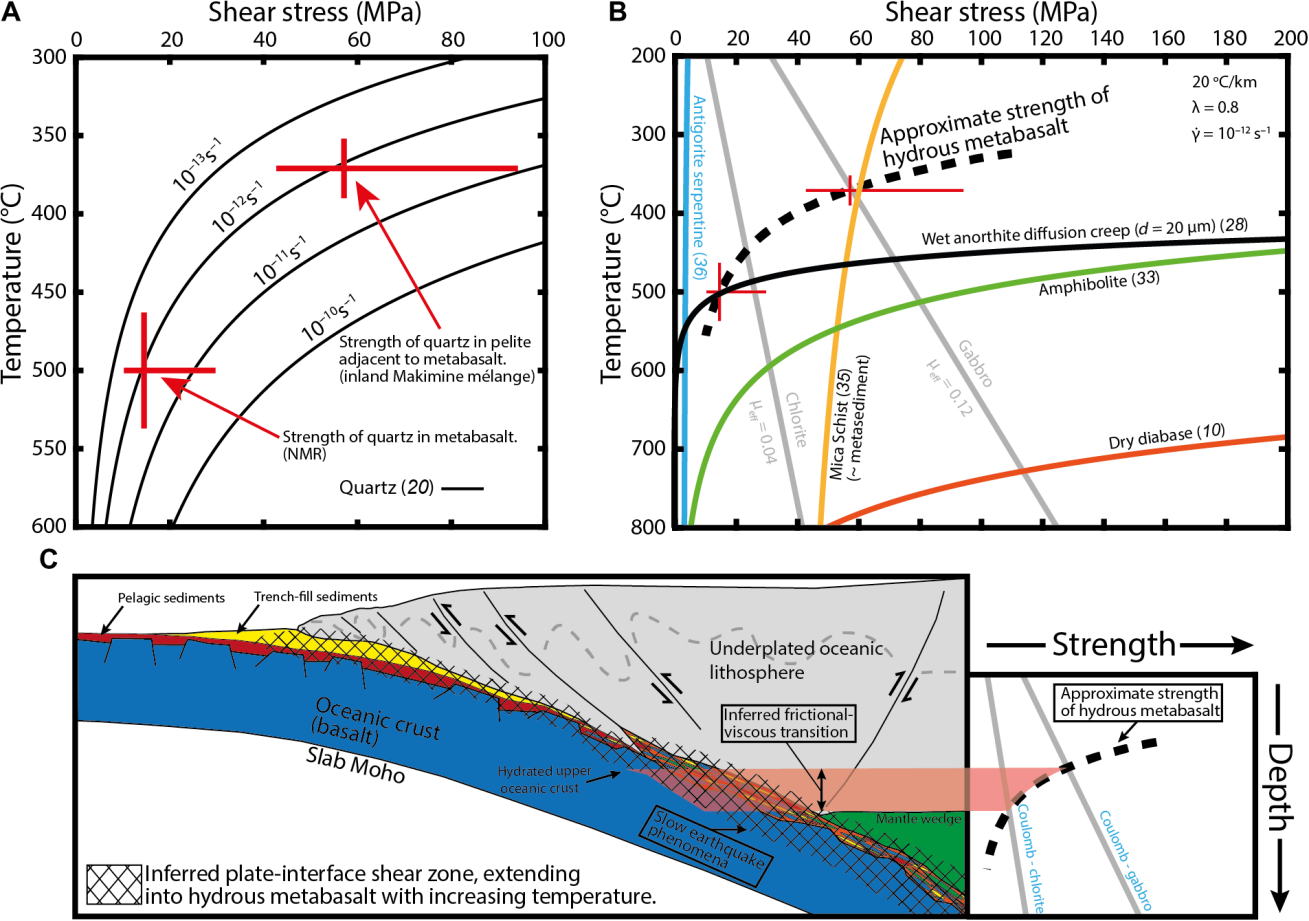
Constraints on the shear strength and deformation temperature of exhumed hydrous oceanic crust and comparison to experimentally determined rheologies for subducting lithologies. (**A**) Piezometer-derived shear stress estimates and RSCM-derived temperature estimates for quartz veins deformed in inland Makimine mélange and the NMR. The experimentally determined shear strength of quartz undergoing dislocation creep (*20*) gives stresses close to the estimated values for the exhumed shear zones at a strain rate of 10^−**12**^ s^−**1**^. (**B**) The piezometer and geothermometer estimates define a strength-temperature curve for hydrous oceanic crust, which is far weaker than dry diabase (*9*). Coulomb strengths with effective friction μ_eff_ = 0.12 and μ_eff_ = 0.04 represent the cohesion-less strength of unaltered oceanic crust (*9*) and chlorite (*13*), respectively (assuming a pore fluid factor λ = 0.8). The sketched strength curve suggests a frictional-viscous transition near ~400°C. The mica schist flow law (*35*) represents the viscous strength of phyllosilicate-rich subducted sediment. (**C**) Cartoon cross section shows the broad plate interface shear zone extending into hydrated oceanic crust, as deformation by solution-transfer creep becomes effective with increasing temperature. As represented here, the frictional-viscous transition in hydrated oceanic crust would occur up-dip of the mantle wedge corner.

Piezometers relate dynamically recrystallized grain sizes to flow stresses during viscous deformation, allowing estimates of the strength of naturally deformed rocks. Quartz veins are present in the inland Makimine metabasalt, but many of the quartz grains are pinned by chlorite, making them unsuitable for piezometry. Using an EBSD-based method (*30*), we calculated a recrystallized quartz grain size of 10.2 ± 5.16 μm from foliation-parallel quartzite layers with pinch-and-swell geometries in inland Makimine metasediment (fig. S2). These grain sizes suggest shear stresses in the range of 43 to 94 MPa. We expect that the strengths obtained from quartzite layers in metasediment are comparable to the strength of metabasalt, given similar pinch-and-swell geometry of quartzite and metabasalt layers within the metasediment and that the amplitude and wavelength of pinch-and-swell structures reflect the magnitude of viscosity contrast (*31*). Boudinaged quartz veins in NMR amphibolite (fig. S3) have microstructures indicating deformation mostly by grain-boundary migration recrystallization. We isolate a population of grains with low internal distortion and a grain size of 70.3 ± 44 μm (fig. S3), giving stresses of 10 to 30 MPa, again using an EBSD-based method (*30*). These stresses are an upper bound on the strength of metabasalt, as the boudinage of quartz veins implies that they are stronger than the host amphibolite. The piezometer-derived strength estimates and RSCM temperature estimates for the NMR and inland Makimine mélange define a strength-temperature curve for the Kyushu metabasalts. Comparison with a quartz rheology (*20*) suggests that the deformation preserved in the Kyushu metabasalts occurred at a strain rate of ~10^−12^ s^−1^ (Fig. 6A), consistent with previous bulk strain rate estimates for margin-scale shear zones (*32*).

### Strength of the oceanic crust

At a strain rate of 10^−12^ s^−1^, approximately equivalent to the strain rate experienced by the Kyushu metabasalts (Fig. 6A), the commonly used flow law for dry diabase (*10*) suggests that unaltered oceanic crust requires temperatures >650°C for shear stresses to be less than <200 MPa. At the same strain rate, a flow law for amphibolite (*33*) requires temperatures >400°C for shear stresses <200 MPa. In contrast, our geologically constrained creep strengths for relatively competent quartzite layers deformed during bulk noncoaxial shear within hydrous metabasalt suggest that deformation of hydrated oceanic crust occurred at shear stresses of approximately 43 to 94 MPa at ~370°C and less than 10 to 30 MPa at ~500°C.

Overall, we observed that phyllosilicates are ubiquitous in the Kyushu metabasalts and well oriented for slip along mechanically weak basal planes. We infer that the presence of interconnected phyllosilicates and water-saturated conditions in the Kyushu metabasalts facilitated solution-precipitation creep and frictional sliding along phyllosilicate laminae (*34*), accommodating macroscopically ductile deformation of the oceanic crust at shear stresses far lower than predicated for unaltered basalt. To illustrate this difference between hydrous metabasalt and unaltered basalt, Fig. 6B compares our geologically constrained ductile shear strengths for metabasalt to existing mechanical data, assuming a strain rate of 10^−12^ s^−1^, a pore fluid factor (pore fluid pressure/vertical stress) λ = 0.8, and a geothermal gradient of 20°C/km. Our geologically constrained strengths are significantly less than predicted by flow laws for dry diabase (*10*) and amphibolite (*33*). The Coulomb strengths of gabbro (*9*) and chlorite (*13*), representing end-member frictional strengths for the oceanic crust, intersect the estimated ductile strength-temperature curve for hydrous metabasalt at shear stresses between ~60 MPa and ~25 MPa at temperatures between ~370° and ~450°C (Fig. 6B), implying a frictional-viscous transition for hydrous metabasalts within these shear stress and temperature ranges.

Subducted phyllosilicate-rich sediments represented by a mica-schist flow law (*35*) have a strength approximately equal to our piezometer estimate at ~370°C but are stronger than our piezometer estimate for metabasalt at ~500°C (Fig. 6B). Our field observations of near-parallel layering of ductilely deformed metasediment and metabasalt in Makimine mélange (Figs. 2A and 3A) suggest that hydrated oceanic crust and metasediment have similar viscosities. Parallel layering of metasediment and amphibolite in the NMR is not as clear because of the limited spatial extent of the outcrop. However, exposed metasediment-amphibolite contacts are approximately planar (Fig. 4A), and we do not observe indications of marked viscosity contrasts between metasediment and amphibolite. Another mineral commonly inferred to be important for subduction thrust shear is antigorite serpentine. The power-law equation for dislocation creep in antigorite (*36*) predicts shear strengths <5 MPa over the entire temperature range considered (Fig. 6B). However, the mechanisms controlling ductile deformation in natural antigorite are uncertain, as is the extrapolation of laboratory behavior to nature (*37*). Direct field observations of scattered lenses of antigorite in NMR amphibolite [see map in (*16*)] suggest that at metamorphic temperatures of ~500°C and geological strain rates, antigorite serpentine may have a greater viscosity than hydrated oceanic crust.

### Implications for active margins

In general, the extent of weakening by growth of phyllosilicates will depend upon their volume fraction and interconnectivity (*38*). The volume fraction of phyllosilicates in metabasalt, and the strain accommodated by dissolution-precipitation creep, will primarily depend on the amount of hydrous fluid available to the crust during subduction and any initial clay content induced by hydration at the mid-ocean ridge, on the sea floor, and at the outer rise. The estimated strengths of the exhumed hydrous metabasalts suggest that if subducting crust is sufficiently hydrated, then creep in metabasalt can accommodate megathrust strain at similar driving stress to weak phyllosilicate-rich sediments, negating the need for a continuous layer of lubricating subducted sediment to explain a low viscosity plate interface at the base of the seismogenic zone. An important depth limitation to extrapolation of this conclusion is that the weakness of metabasalt will only apply at pressure, temperature, and fluid conditions unsuitable for pervasive eclogitization.

Taking the frictional strengths of chlorite (*13*) and gabbro (*9*) as lower and upper bounds on the frictional strength of the oceanic crust, and assuming a thermal gradient of 20°C/km and a nominal pore fluid factor of λ = 0.8, the frictional-viscous transition for hydrated oceanic crust is expected to occur in the range of 370° to 450°C at shear stresses between 60 MPa and 25 MPa (Fig. 6, B and C). On this basis, we suggest that the frictional-viscous transition in hydrated oceanic crust may influence the bulk rheology and slip behavior of the subduction thrust interface in this temperature range. Lateral variations in creep versus seismogenic slip may also be explained by variations in basalt rheology. Faulting on the sea floor and at the outer rise is likely to increase permeability, allowing locally pervasive hydration of the oceanic crust. Given that hydration may vary from margin to margin, and within margins, the strength of the basaltic crust may be quite variable. We note that if the basaltic crust is hydrous and foliated, then the low velocity seismic signals that are commonly interpreted as the sediment-dominated megathrust may also originate from hydrated oceanic crust. Overall, this study provides a field and microstructural basis to recent margin-scale observations in Alaska (*39*), where it has been suggested that plate interface creep occurs in areas where the oceanic crust is more hydrated. Weakening of the oceanic crust because of hydration may be a common process along all creeping margins. We suspect that, along colder margins, our observations may be applicable at greater depths, because the deformation mechanisms we infer are largely temperature (rather than pressure) sensitive.

## CONCLUSION

Coastal Makimine mélange, inland Makimine mélange, and NMR record Late Cretaceous plate boundary deformation at the Eurasian margin. Peak metamorphic temperatures from 300° to 500°C suggest that these three shear zones are exhumed analogs for deformation at and below the base of a megathrust seismogenic zone. In all three shear zones, metabasalt contains aligned and interconnected phyllosilicate minerals surrounding more competent minerals deformed by diffusion creep with dominant shear sense consistent with paleo-subduction kinematics. Paleo-strength estimates using recrystallized quartz grain-size piezometry support the hypothesis of weak hydrous metabasalts; calculated strengths for the exhumed metabasalts are comparable to metasediments and are significantly weaker than experimentally determined flow laws for unaltered diabase. In general, the results suggest that the strength of hydrous oceanic crust is weak enough to control subduction thrust rheology in the viscous regime at the base of the seismogenic zone. Hydrous metabasalts may therefore form a substantial part of the weak, tabular zones commonly inferred to be present along subduction interfaces, instead of or in addition to the metasediments typically thought to make up this layer. Consequently, the hydration state of oceanic crust may have a profound control on the depth of interseismic coupling, particularly in relatively sediment-starved margins.

## MATERIALS AND METHODS

### Electron microscopy

EDS and EBSD maps were produced from thin sections polished using colloidal silica and coated with 5 to 8 nm of carbon to prevent charging. Data were acquired using a Zeiss Sigma HD Field Emission Gun Analytical SEM fitted with two Oxford Instruments 150-mm^2^ energy-dispersive X-ray spectrometers and a Nordlys EBSD system with Oxford Instruments Aztec software. During EDS mapping, the SEM was operated at an accelerating voltage of 20 kV with a nominal beam current of 4.3 nA. During EBSD mapping, the SEM was operated in high-current mode (8.5 nA) with an accelerating voltage of 20 kV, and the sample was inclined at 70° to the incident beam. EBSD data were processed using the MTEX toolbox for MATLAB (*40*).

### Strength calculations

To obtain shear strength estimates, we require estimates of temperature, normal stress, and fluid pressure as a function of depth. To obtain temperature, we impose a 20°C/km geothermal gradient. To derive normal stress, we assume a gently dipping subduction thrust interface, where σ_n_ can be approximated as the vertical (lithostatic) stress σ_v_

$$\sigma_{n}\approx \sigma_{v}=\rho gz$$

where ρ is the density of the subduction thrust hanging wall, assumed to be 2650 kg/m^3^; *g* is acceleration due to gravity; and *z* is depth. Frictional simple shear strengths (τ_f_) are determined by

$$\tau_{f}=\sigma_{n*}\mu_{\text{eff}}$$

where μ_eff_ is the effective friction coefficient determined by

$$\mu_{\text{eff}}=\mu_{f*}(1-\lambda )$$

where λ is the pore fluid factor relating pore fluid pressure (*P*_f_) and vertical stress

$$\lambda =P_{f}/\sigma_{v}$$

Viscous simple shear strengths (τ_v_) are determined from the flow laws mentioned in the text, adapted to simple shear following (*41*)

$$\tau_{v}={\left(\frac{\dot{\gamma }}{A*\sqrt{\left(3^{n+1}\right)}*{\left(f_{H_{2}O}\right)}^{r}*e^{\frac{-Q}{\mathrm{RT}}}}\right)}^{\frac{1}{n}}$$

where $\dot{\gamma }$ is the strain rate; *A*, *n*, *r*, and *Q* are experimentally determined parameters; *f*_H_2_O_ is the water fugacity; *R* is the gas constant; and *T* is the temperature in Kelvin. The relationship between *f*_H_2_O_, pressure, and temperature is fitted with an Arrhenius relationship following (*42*)

$$f_{H_{2}O}=a_{H_{2}O}*A_{1}e^{\frac{\left(-A_{2}+P*A_{3}\right)}{R*T}}$$

where *a*_H_2_O_ is water activity (assumed to be 0.999); *P* is pressure (Pa), which we assume to be lithostatic; and *A*_1_, *A*_2_, and *A*_3_ are empirically fitted constants with the values 5521 MPa, 31,280 J mol^−1^, and −2.009 × 10^−5^ m^3^.

Paleo-differential stress estimates were obtained from EBSD maps of dynamically recrystallized quartz using the 1-μm step size piezometer of (*30*). Shear stresses were assumed to be approximated as half of the differential stresses.

## Supplementary Material

aba1529_SM.pdf

## SUPPLEMENTARY MATERIALS

Supplementary material for this article is available at http://advances.sciencemag.org/cgi/content/full/6/22/eaba1529/DC1
