# Supplementary material for: Hydrous oceanic crust hosts megathrust creep at low shear stresses
Source: Sci Adv. 2020 May 27;6(22):eaba1529. doi: 10.1126/sciadv.aba1529 (PMC7253158; doi:10.1126/sciadv.aba1529)
Supplement: aba1529_SM.pdf [file aba1529_SM.pdf]

Science Advances
AAAS

advances.sciencemag.org/cgi/content/full/6/22/eaba1529/DC1

Supplementary Materials for

Hydrous oceanic crust hosts megathrust creep at low shear stresses

Christopher J. Tulley*, Åke Fagereng, Kohtaro Ujiie

*Corresponding author. Email: tulleycj@cardiff.ac.uk

Published 27 May 2020, *Sci. Adv.* **6**, eaba1529 (2020)
DOI: 10.1126/sciadv.aba1529

This PDF file includes:

Figs. S1 to S3
References

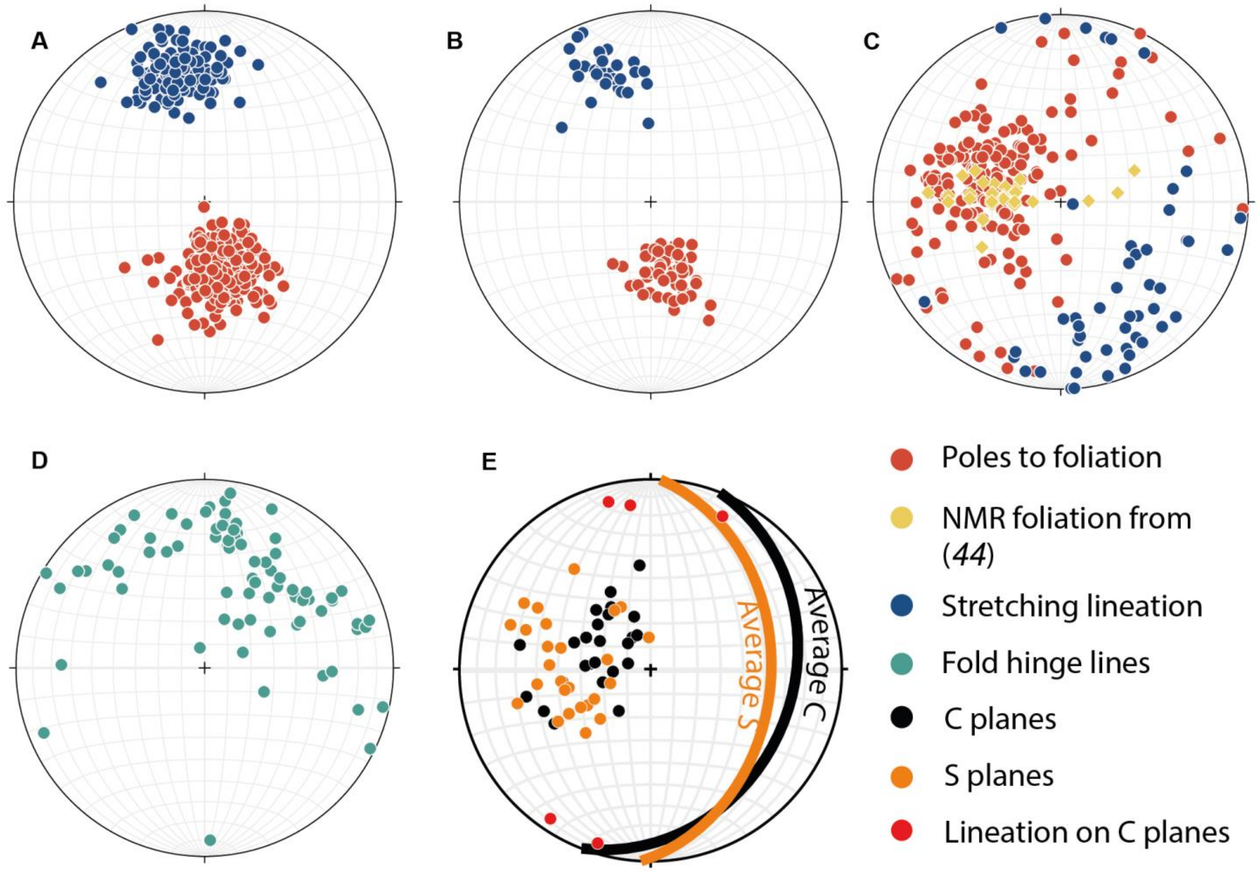

Supplementary Figure S1 Structural measurements from Makimine mélange and the NMR. (A) Foliation and lineation in coastal Makimine mélange. (B) Foliation and lineation in inland Makimine mélange. (C) Foliation, including a regional dataset (*44*) and stretching lineation from the NMR. (D) Fold hinge lines in metabasalt and metapelite from the NMR. (E) S and C planes measured in chlorite-actinolite schists of the NMR.

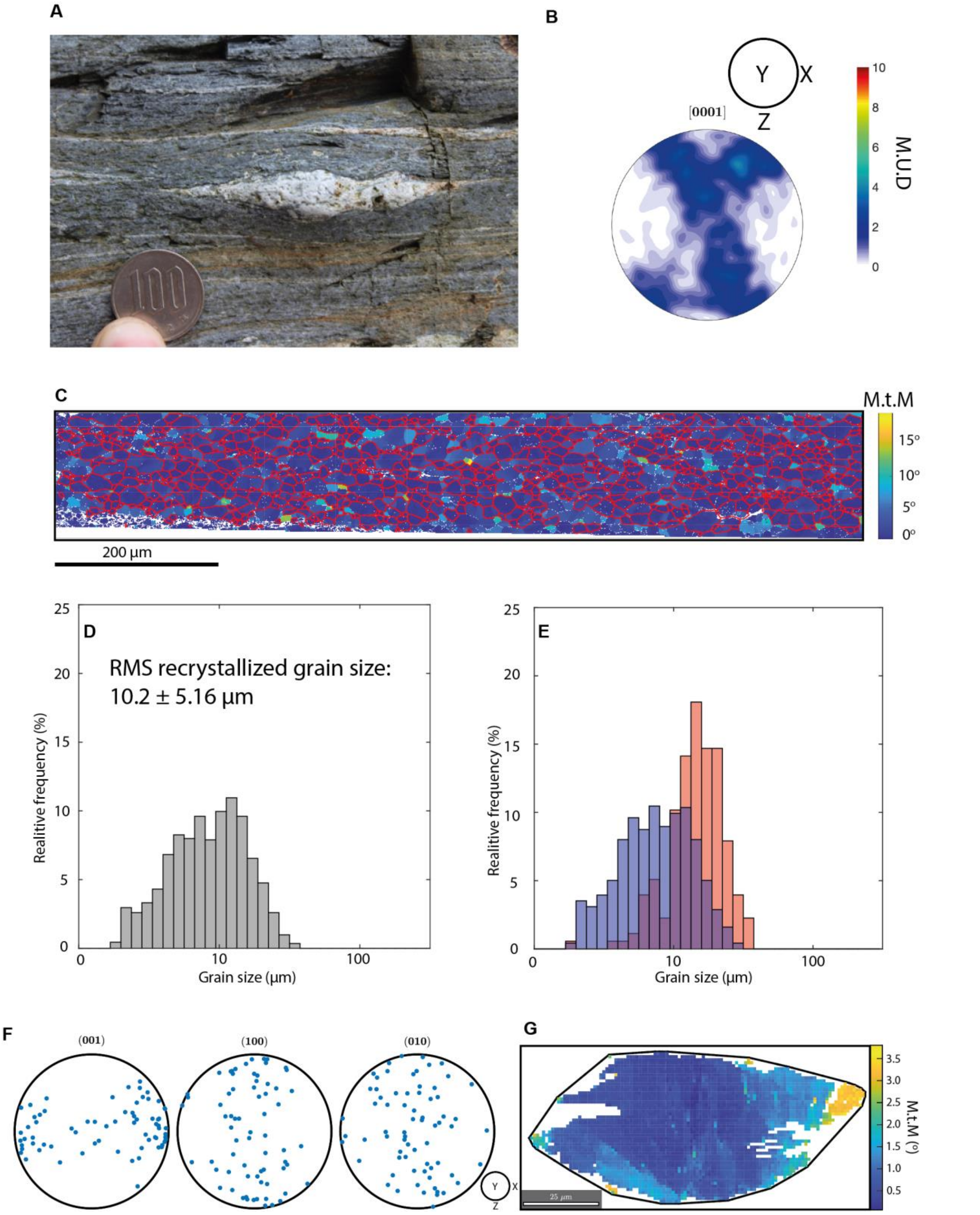

Supplementary Figure S2 EBSD data collected from deformed quartz and clinopyroxene in inland Makimine mélange. (A) Boudinaged quartzite lens and pinch and swell in quartzite layers within inland Makimine mélange metasediment. (B) Quartz C axis pole figure constructed from data in map (C). EBSD map of recrystallized quartz in pelite from inland Makimine mélange. Pixels are contoured by their misorientation with respect to the mean orientation of their parent grain (misorientation to mean orientation, M.t.M).

Recrystallized grains (red boundaries) have low M.t.M values indicating low degrees of internal distortion. (D) Histogram of recrystallized quartz grain size, excluding grains that bisect the map border. (E) Histogram of all quartz grains, excluding grains that bisect the map border. Relict grains are plotted in red, recrystallized in blue. (F) Pole figures showing the orientation of 69 pyroxene grains from inland Makimine metabasalt. There is a weak [001] preferred orientation. (G) EBSD misorientation map (M.t.M) of a pyroxene grain, internal distortion is generally < 2 ^{o}. Photo credit (A); C. Tulley, Cardiff University.

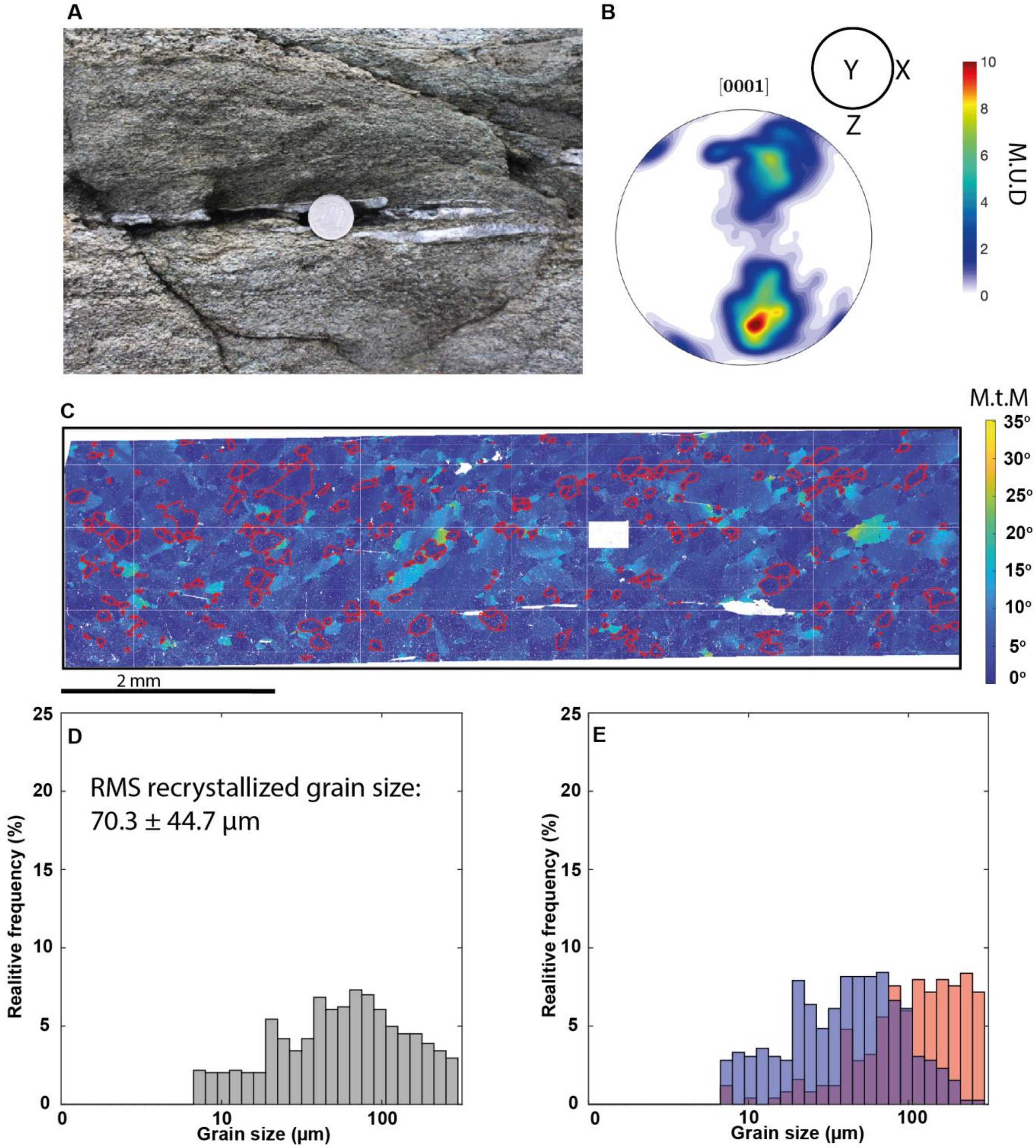

Supplementary Figure S3. EBSD data collected from a quartz vein deformed in amphibolite from the NMR.

(A) Quartz vein deformed parallel to the metamorphic foliation in amphibolite from the NMR. B) Quartz C

axis pole figure constructed from data in map (C). (C) EBSD map of recrystallized quartz from a boundinaged vein. Pixels are contoured by their misorientation with respect to the mean orientation of their parent grain (misorientation to mean orientation, M.t.M). Recrystallized grains (red boundaries) have low M.t.M values indicating low degrees of internal distortion. The white square outlines a frame which failed to acquire any data. (D) Histogram of recrystallized grain size, excluding grains that bisect the map border. (E) Histogram of all grains, excluding grains that bisect the map border. Relict grains are plotted in red, recrystallized in blue. Photo credit (A); C. Tulley, Cardiff University.

REFERENCES

1. G. A. Abers, Seismic low-velocity layer at the top of subducting slabs: Observations, predictions, and systematics. *Phys. Earth Planet. Inter.* **149**, 7–29 (2005).

2. R. L. Shreve, M. Cloos, Dynamics of sediment subduction, melange formation, and prism accretion. *J. Geophys. Res.* **91**, 10229–10245 (1986).

3. W. M. Behr, T. W. Becker, Sediment control on subduction plate speeds. *Earth Planet. Sci. Lett.* **502**, 166–173 (2018).

4. S. V. Sobolev, M. Brown, Surface erosion events controlled the evolution of plate tectonics on Earth. *Nature* **570**, 52–57 (2019).

5. S. M. Peacock, Fluid processes in subduction zones. *Science*, **248**, 329–337 (1990).

6. J. C. Moore, P. Vrolijk, Fluids in accretionary prisms. *Rev. Geophys.* **30**, 113–135 (1992).

7. K. Wang, S. L. Bilek, Do subducting seamounts generate or stop large earthquakes? *Geology* **39**, 819–822 (2011).

8. B. W. Tichelaar, L. J. Ruff, Depth of seismic coupling along subduction zones. *J. Geophys. Res. Solid Earth*. **98**, 2017–2037 (1993).

9. C. He, Z. Wang, W. Yao, Frictional sliding of gabbro gouge under hydrothermal conditions. *Tectonophysics* **445**, 353–362 (2007).

10. S. J. Mackwell, M. E. Zimmerman, D. L. Kohlstedt, High-temperature deformation of dry diabase with application to tectonics on Venus. *J. Geophys. Res. Solid Earth* **103**, 975–984 (1998).

11. J. L. Hardebeck, J. P. Loveless, Creeping subduction zones are weaker than locked subduction zones. *Nat. Geosci.* **11**, 60–64 (2018).

12. Å. Fagereng, J. Diener, S. Ellis, F. Remitti, Fluid-related deformation processes at the up- and down-dip limits of the subduction thrust seismogenic zone: What do the rocks tell us?, in *Geology and Tectonics of Subduction Zones: A Tribute to Gaku Kimura*, T. Byrne, M. B. Underwood, D. Fisher, L. McNeill, D. Saffer, K. Ujiie, A. Yamaguchi, Eds. (The Geological Society of America, 2018), vol. 534, pp. 1–30.

13. A. S. Okamoto, B. A. Verberne, A. R. Niemeijer, M. Takahashi, I. Shimizu, T. Ueda, C. J. Spiers, Frictional properties of simulated chlorite gouge at hydrothermal conditions: Implications for subduction megathrusts. *J. Geophys. Res. Solid Earth* **124**, 4545–4565 (2019).

14. V. M. Mares, A. K. Kronenberg, Experimental deformation of muscovite. *J. Struct. Geol.* **15**, 1061–1075 (1993).

15. K. Ujiie, H. Saishu, Å. Fagereng, N. Nishiyama, M. Otsubo, H. Masuyama, H. Kagi, An explanation of episodic tremor and slow slip constrained by crack-seal veins and viscous shear in subduction mélange. *Geophys. Res. Lett.* **45**, 5371–5379 (2018).

16. Y. Mori, M. Shigeno, K. Miyazaki, T. Nishiyama, Peak metamorphic temperature of the Nishisonogi unit of the Nagasaki metamorphic rocks, western Kyushu, Japan. *J. Mineral. Petrol. Sci.* **114**, 170–177 (2019).

17. H. Hara, K. Kimura, Metamorphic and cooling history of the Shimanto accretionary complex, Kyushu, Southwest Japan: Implications for the timing of out-of-sequence thrusting. *Isl. Arc*. **17**, 546–559 (2008).

18. K. Miyazaki, M. Ozaki, M. Saito, S. Toshimitsu, in *The Geology of Japan*, T. Moreno, S. R. Wallis, T. Kojima, W. Gibbon, Eds. (The Geological Society, 2016), pp.139–174.

19. J. M. Whittaker, R. D. Müller, G. Leitchenkov, H. Stagg, M. Sdrolias, C. Gaina, A. Goncharov, Major Australian-Antarctic plate reorganization at Hawaiian-Emperor bend time. *Science*, **318**, 83–86 (2007).

20. G. Hirth, C. Teyssier, J. W. Dunlap, An evaluation of quartzite flow laws based on comparisons between experimentally and naturally deformed rocks. *Int. J. Earth Sci.* **90**, 77–87 (2001).

21. K. Ujiie, K. Noro, N. Shigematsu, Å. Fagereng, N. Nishiyama, C. J. Tulley, H. Masuyama, Y. Mori, Geological and rheological conditions of subduction plate boundary between the seismogenic zone and the ETS zone in warm-slab environments. *Japan Geosci. Union Meet.* (2019).

22. G. Palazzin, H. Raimbourg, V. Famin, L. Jolivet, Y. Kusaba, A. Yamaguchi, Deformation processes at the down-dip limit of the seismogenic zone: The example of Shimanto accretionary complex. *Tectonophysics* **687**, 28–43 (2016).

23. S. M. Schmid, M. Casey, in *Geophysical Monograph Series* (American Geophysical Union, 1986), pp. 263–286.

24. E. D. Dempsey, D. J. Prior, E. Mariani, V. G. Toy, D. J. Tatham, Mica-controlled anisotropy within mid-to-upper crustal mylonites: An EBSD study of mica fabrics in the Alpine Fault Zone, New Zealand. *Geol. Soc. Lond. Spec. Publ.* **360**, 33–47 (2011).

25. S. Wassmann, B. Stöckhert, Rheology of the plate interface—Dissolution precipitation creep in high pressure metamorphic rocks. *Tectonophysics* **608**, 1–29 (2013).

26. E. H. Rutter, D. Elliott, The kinetics of rock deformation by pressure solution [and discussion]. *Philos. Trans. R. Soc. A Math. Phys. Eng. Sci.* **283**, 203–219 (1976).

27. Y. Mori, M. Shigeno, T. Nishiyama, Fluid-metapelite interaction in an ultramafic mélange: Implications for mass transfer along the slab-mantle interface in subduction zones. *Earth Planets Space* **66**, 47 (2014).

28. E. Rybacki, G. Dresen, Dislocation and diffusion creep of synthetic anorthite aggregates. *J. Geophys. Res. Solid Earth* **105**, 26017–26036 (2000).

29. A. Beall, Å. Fagereng, S. Ellis, Strength of strained two-phase mixtures: Application to rapid creep and stress amplification in subduction zone mélange. *Geophys. Res. Lett.* **46**, 169–178 (2019).

30. A. J. Cross, D. J. Prior, M. Stipp, S. Kidder, The recrystallized grain size piezometer for quartz: An EBSD-based calibration. *Geophys. Res. Lett.* **44**, 6667–6674 (2017).

31. J. G. Ramsay, *Folding and Fracturing of Rocks* (McGraw-Hill, 1967).

32. Å. Fagereng, J. Biggs, New perspectives on 'geological strain rates' calculated from both naturally deformed and actively deforming rocks. *J. Struct. Geol.* **125**, 100–110 (2019).

33. B. R. Hacker, J. Christie, Brittle/ductile and plastic/cataclastic transitions in experimentally deformed and metamorphosed amphibolite. *Geophys. Monogr.* **56**, 127–147 (1990).

34. B. Bos, C. J. Spiers, Frictional-viscous flow of phyllosilicate-bearing fault rock: Microphysical model and implications for crustal strength profiles. *J. Geophys. Res.* **107**, ECV1-1–ECV1-13 (2002).

35. W. T. Shea, A. K. Kronenberg, Rheology and deformation mechanisms of an isotropic mica schist. *J. Geophys. Res.* **97**, 15201–15237 (1992).

36. N. Hilairet, B. Reynard, Y. Wang, I. Daniel, S. Merkel, N. Nishiyama, S. Petitgirard, High-pressure creep of serpentine, interseismic deformation, and initiation of subduction. *Science* **318**, 1910–1913 (2007).

37. A.-L. Auzende, J. Escartin, N. P. Walte, S. Guillot, G. Hirth, D. J. Frost, Deformation mechanisms of antigorite serpentinite at subduction zone conditions determined from experimentally and naturally deformed rocks, *Earth Planet Sci. Lett.* **411**, 229–240 (2015).

38. M. R. Handy, The solid-state flow of polymineralic rocks. *J. Geophys. Res.* **95**, 8647–8661 (1990).

39. D. J. Shillington, A. Bécel, M. R. Nedimović, H. Kuehn, S. C. Webb, G. A. Abers, K. M. Keranen, J. Li, M. Delescluse, G. A. Mattei-Salicrup, Link between plate fabric, hydration and subduction zone seismicity in Alaska. *Nat. Geosci.* **8**, 961–964 (2015).

40. F. Bachmann, R. Hielscher, H. Schaeben, Texture analysis with MTEX—Free and open source software toolbox. *Solid State Phenom.* **160**, 63–68 (2010).

41. J. F. Nye, The flow law of ice from measurements in glacier tunnels, laboratory experiments and the Jungfraufirn borehole experiment. *Proc. R. Soc. London. Ser. A. Math. Phys. Sci.* **219**, 477–489 (1953).

42. W. J. Shinevar, M. D. Behn, G. Hirth, Compositional dependence of lower crustal viscosity. *Geophys. Res. Lett.* **42**, 8333–8340 (2015)

43. Y. Isozaki, K. Aoki, T. Nakama, S. Yanai, New insight into a subduction-related orogen: A reappraisal of the geotectonic framework and evolution of the Japanese Islands. *Gondw. Res.* **18**, 82–105 (2010).

44. T. Nishiyama, Petrologic study of the Nagasaki metamorphic rocks in the Nishisonogi peninsula – with special reference to the greenrock complex and the reaction-enhanced ductility. *Mem. Geol. Soc. Japan* **33**, 237–257 (1989).
